# How different is the care of terminal pancreatic cancer patients in inpatient palliative care units and acute hospital wards? A nationwide population-based study

**DOI:** 10.1186/s12904-016-0075-x

**Published:** 2016-01-08

**Authors:** Jack P. Wang, Chen-Yi Wu, I-Hsuan Hwang, Chien-Hui Kao, Yi-Ping Hung, Shinn-Jang Hwang, Chung-Pin Li

**Affiliations:** Division of Gastroenterology and Hepatology, Department of Medicine, Taipei Veterans General Hospital, No. 201, Sec. 2, Shih-Pai Road, Taipei, 11217 Taiwan; National Yang-Ming University School of Medicine, Taipei, Taiwan; Department of Internal Medicine, Taipei City Hospital, Renai Branch, Taipei, Taiwan; Department of Dermatology, Taipei Veterans General Hospital, No. 201, Sec. 2, Shih-Pai Road, Taipei, 11217 Taiwan; Institute of Public Health & Department of Public Health, National Yang-Ming University, Taipei, Taiwan; College of Healthcare Administration and Management, National Taipei University of Nursing and Health Sciences, Taipei, Taiwan; Department of Oncology, Taipei Veterans General Hospital, Taipei, Taiwan; Department of Family Medicine, Taipei Veterans General Hospital, Taipei, Taiwan

**Keywords:** End-of-life care, Pancreatic cancer, Hospice, Inpatient palliative care

## Abstract

**Background:**

Inpatient palliative care is important for patients with terminal pancreatic cancer. However, the differences between inpatient palliative care and acute hospital care for inpatients with pancreatic cancer have not been explored in a population-based study.

**Methods:**

This population-based nationwide study was conducted using data from the Taiwan National Health Insurance database to analyze the differences between inpatient palliative care and acute hospital care for inpatients with pancreatic cancer. We identified 854 patients with terminal pancreatic cancer, who had received in-hospital end-of-life care between January 2003 and December 2006. These patients were then sub-divided and matched 1:1 (using propensity score matching) according to whether they received inpatient palliative care (*n* = 276) or acute hospital care (*n* = 276). These groups were subsequently compared to evaluate any differences in the use of aggressive procedures, prescribed medications, and medical costs.

**Results:**

Inpatient palliative care was typically provided by family physicians (39 %) and oncologists (25 %), while acute hospital care was typically provided by oncologists (29 %) and gastroenterologists (24 %). The inpatient palliative care group used natural opium alkaloids significantly more frequently than the acute hospital care group (84.4 % vs. 56.5 %, respectively; *P* < 0.001). The inpatient palliative care group also had shorter hospital stays (10.6 ± 11.1 days vs. 20.6 ± 16.3 days, respectively; *P* < 0.001), fewer aggressive procedures, and lower medical costs (both, *P <* 0.005).

**Conclusions:**

Compared to patients in acute hospital wards, patients with pancreatic cancer in inpatient palliative care units received more frequent pain control treatments, underwent fewer aggressive procedures, and incurred lower medical costs. Therefore, inpatient palliative care should be considered a viable option for patients with terminal pancreatic cancer.

## Background

Pancreatic cancer is one of the worldwide leading causes of cancer-related death [1], and pancreatic cancer is the fourth most common type of solid tumor in the United States [1]. Unfortunately, most patients with pancreatic cancer are in the advanced stages, and have a median expected survival period of <8 months [2]. In addition, patients with terminal pancreatic cancer frequently suffer from weight loss, jaundice, loss of appetite, nausea, vomiting, abdominal pain, back pain, cachexia, esophageal variceal bleeding, and ascites [2]. Therefore, symptomatic treatment is important for relieving the discomfort that is experienced by these patients.

Hospice care aims to provide supportive care to patients who are in the final stage of a terminal illness [3]. Supportive care is the treatment given to prevent, control, or relieve complications and side effects and to improve the patient's comfort and quality of life [4]. Therefore, hospice care focuses on improving the patient’s comfort and quality of life, rather than achieving a cure for their condition. Hospice programs typically use a multidisciplinary approach, which includes the services of doctors, nurses, social workers, and clergy, in order to offer holistic care to patients. Based on this comprehensive care, it has been reported that patients who receive hospice care experience a better quality of life, compared to patients with similar conditions who receive conventional care [5].

Palliative care is an approach that improves the quality of life of patients and their families facing the problems that are associated with life-threatening illness, through the prevention and relief of suffering by early identification and impeccable assessment and treatment of pain and other physical, psychosocial, and spiritual problems [6]. Inpatient palliative care in Taiwan is covered by National Health Insurance (NHI) programs, and is offered by many medical centers (medical facilities that are larger than regional or district hospitals) or school-affiliated hospitals. However, only a small percentage of terminally ill patients ultimately select inpatient palliative care [7, 8], and the rate of inpatient palliative care use was 12.3 % among patients with cancer who died between 2000 and 2004 [8]. Furthermore, although the trend of inpatient palliative care use increased from 5.5 % in 2000 to 15.4 % in 2004, the rate of Taiwanese inpatient palliative care use remains far below the rates in Western countries [7–10]. This relatively low usage may be due to individual misconceptions, physician preferences, and/or cultural concerns [9, 10]. A previous study in a Taiwanese inpatient palliative care unit reported that inpatients with advanced cancer in inpatient palliative care units had a shorter length of stay, compared to inpatients who were treated at acute hospital wards [11]. Nevertheless, there are no studies that have performed a comprehensive, nationwide comparison of inpatient palliative care and acute hospital care for patients with pancreatic cancer. Therefore, using information from Taiwan’s NHI database, we aimed to compare the patient characteristics, medical procedures, prescriptions, and medical costs for patients with pancreatic cancer who received inpatient palliative care or acute hospital care, and to identify any significant differences between these groups.

## Methods

### Data sources

Taiwan’s NHI program is a compulsory universal health insurance program (approximately 97 % of all residents were enrolled in 2004), which offers comprehensive medical care coverage to all residents. The NHI dataset, which is compiled and managed by the National Health Research Institutes and publicly available to researchers, consists of comprehensive healthcare information from all NHI beneficiaries [12].

In this study, we obtained records, including medical order files and hospital administration files, from all inpatient beneficiaries who made a claim between January 2003 and December 2006. Using these records, we extracted anonymized inpatient identification numbers, dates of admission and discharge, 1 major and 4 minor disease ICD-9-CM diagnoses (International Classification of Diseases, 9th Revision, Clinical Modification), examination costs, procedures, surgeries, prescribed drugs, and special medical materials that were used during the admission. The patients’ medical order files also contained details regarding the doctors’ orders and laboratory tests. The accreditation level of each hospital (i.e., medical center, regional hospital, or district hospital) was determined using the hospital’s administration files. A complete database of the coding numbers, which identifies the prescribed drugs and procedures, were obtained from the NHI website [13].

This study was conducted in accordance with the Helsinki Declaration, and was approved by the institutional review board of National Yang-Ming University (approval no. 1000046).

### Study population

In this study, we identified 854 pancreatic cancer patients who were admitted and died in-hospital between January 2003 and December 2006. Pancreatic cancer was defined using an ICD-9-CM compatible code (157) from the Registry for Catastrophic Illness Patient database, which is a separate subsection of the NHI database. Most patients who are diagnosed with cancer sign up for a Catastrophic Illness Card in Taiwan, and these cardholders are exempt from cost-sharing under the NHI program. The effects of comorbidities were estimated using the Charlson comorbidity index [14]. Propensity score matching was performed to minimize the potential influence of selection bias, whereby logistic regression was used to create a propensity score for the inpatient palliative care and acute hospital care groups [15]. The covariates that we examined included patient characteristics (age and sex), Charlson comorbidity index, hospital location, and hospital accreditation level. One-to-one matching was subsequently performed for the two patients groups, using the nearest-neighbor matching method.

### Statistical analysis

Data linkage, processing, and computation were performed using Microsoft Structured Query Language Server 2008 (Microsoft Corp., Redmond, WA, USA), and SPSS software (version 21.0, SPSS Inc., Chicago, IL, USA) was used to analyze the data for descriptive and inferential statistics. The Mann-Whitney *U* test was used to analyze numerical data between the groups, and the chi-square test was used to compare categorical variables. A generalized linear model with gamma distribution and a log link function was used to analyze medical costs, which are typically right-skewed [16]. Differences were considered statistically significant at a *P*-value of <0.05. All data were presented as mean ± standard deviation or number (%) as appropriate.

## Results

### Baseline characteristics

Among the 854 terminally ill patients with pancreatic cancer that we identified, 276 (32 %) patients were treated in inpatient palliative care units (173 men and 103 women; mean age = 68.6 years) and 578 (68 %) were treated at acute hospital wards (354 men and 224 women; mean age = 67.5 years) (Table 1). All physicians who treated these patients had passed standardized hospice specialist training courses in Taiwan. The inpatient palliative care group was typically managed by family physicians (39 %) and oncologists (25 %), whereas the acute hospital care group was typically managed by internal medicine physicians (39 %, *P* < 0.001), who were generally gastroenterologists (24 %). Significant differences between the palliative care and pre-matched acute care patients were observed for the hospital accreditation level (*P* = 0.03) and hospital regional location (*P* = 0.021). The Charlson comorbidity indices were not significantly different between the two groups, although the length of hospital stay was significantly shorter for the inpatient palliative care group (10.6 ± 11.1 days vs. 20.6 ± 16.7 days, respectively; *P* < 0.001). After one-to-one propensity score matching, 276 matched controls were selected, and we found that the baseline patient characteristics in the two groups were similar (Table 1).

**Table 1:** Baseline characteristics of terminal pancreatic cancer patients according to end-of-life care

	Inpatient palliative care	Acute hospital care			
Characteristics		Pre-matched		Post-matched	
	n (%)	n (%)	*P*	n (%)	*P*
Number of patients	276 (100)	578 (100)		276 (100)	
Age (years; mean ± SD)	68.6 ± 13.4	67.5 ± 12.8	0.265	68.5 ± 12.4	0.953
Sex			0.687		0.535
Male	173 (63)	354 (61)		180 (65)	
Female	103 (37)	224 (39)		96 (35)	
Physicians’ specialty			<0.001		<0.001
Family medicine	108 (39)	38 (7)		24 (7)	
Oncology	69 (25)	174 (30)		77 (29)	
Internal medicine	64 (23)	226 (39)		104 (43)	
General	57 (21)	64 (11)		28 (10)	
Gastroenterology	3 (1)	138 (24)		65 (24)	
Cardiology	0 (0)	3 (1)		2 (0.7)	
Chest medicine	0 (0)	14 (2)		8 (3)	
Nephrology	1 (0.4)	2 (0.3)		0 (0)	
Endocrinology	0 (0)	4 (0.7)		1 (0.4)	
Infection	3 (1)	1 (0.2)		0 (0)	
Radiation oncology	25 (9)	17 (3)		6 (2)	
Surgery	0 (0)	113 (20)		57 (18)	
Neurology	9 (3)	6 (1)		3 (1)	
Others	1 (0.4)	4 (0.7)		4 (1)	
Accreditation level of hospital			0.030		0.304
Medical centers	161 (58)	316 (55)		156 (57)	
Regional hospitals	73 (26)	199 (34)		87 (32)	
District hospitals	42 (15)	63 (11)		33 (12)	
Region of hospital			0.021		0.668
North	171 (62)	401 (69)		171 (62)	
Central	20 (7)	49 (8)		25 (9)	
South	75 (27)	120 (21)		74 (27)	
East	10 (4)	8 (1)		6 (2)	
Charlson comorbidity index (mean ± SD)	6.8 ± 6.5	6.7 ± 6.5	0.798	6.9 ± 2.8	0.564
Length of hospital stay (day, mean ± SD)	10.6 ± 11.1	20.6 ± 16.7	<0.001	20.6 ± 16.3	<0.001

*SD* standard deviation

### Aggressive procedures

The aggressive procedures that were used to treat the inpatient palliative care group and the matched acute hospital care group are shown in Table 2. The patients in the acute hospital care group underwent significantly more aggressive procedures, including urinary catheterization, nasogastric tube feeding, central venous catheter insertion, intensive care unit admission, endotracheal intubation, abdominal drainage, cardiopulmonary resuscitation, total parenteral nutrition, percutaneous transhepatic cholangiography and drainage, chest tapping or intubation, and defibrillation/cardioversion (all *P* < 0.001). Only one patient in the inpatient palliative care group underwent an aggressive procedure (hemodialysis).

**Table 2:** Aggressive procedures used to treat terminal pancreatic cancer patients according to end-of-life care

Procedures	Inpatient palliative care		Acute hospital care		*P*
	(*n* = 276)		(*n* = 276)		
	n	%	n	%	
Urinary catheterization	0	0	156	56.5	<0.001
Nasogastric tube feeding	0	0	86	31.2	<0.001
Central venous catheter insertion	0	0	77	27.9	<0.001
Intensive care unit admission	0	0	57	20.7	<0.001
Endotracheal intubation	0	0	49	17.8	<0.001
Abdominal drainage	0	0	33	12.0	<0.001
Cardiopulmonary resuscitation	0	0	28	10.1	<0.001
Total parenteral nutrition	0	0	25	9.1	<0.001
Percutaneous transhepatic cholangiography and drainage	0	0	23	8.3	<0.001
Chest tapping or intubation	0	0	15	5.4	<0.001
Defibrillation/cardioversion	0	0	9	3.3	<0.001
Hemodialysis	1	0.4	7	2.5	0.068
Epinephrine injection	0	0	4	1.4	0.124

### Drug prescription patterns

The prescription patterns for both care groups are shown in Table 3, according to their Anatomical Therapeutic Chemical classification. In the inpatient palliative care group, the most commonly prescribed medication was natural opium alkaloids (84.4 %), followed by solutions that affect electrolyte balance (80.1 %), propulsives (55.1 %), contact laxatives (52.9 %), and benzodiazepine derivatives (48.2 %). In the matched acute hospital care group, the most commonly prescribed medication was solutions that affect electrolyte balance (97.1 %), followed by solutions for parenteral nutrition (80.8 %), propulsives (72.5 %), plain sulfonamide diuretics (71.7 %), and electrolyte solutions (66.7 %). Compared to the acute hospital care group, the inpatient palliative care patients more frequently received natural opium alkaloids (84.4 % vs. 56.5 %; *P* < 0.001) and benzodiazepine derivatives (48.2 % vs. 26.8 %; *P* < 0.001), although they less frequently received cephalosporins (32.2 % vs. 60.5 %; *P* < 0.001) and adrenergic or dopaminergic agents (5.8 % vs. 52.9 %; *P* < 0.001).

**Table 3:** Medications used to treat terminal pancreatic patients according to end-of-life care

Ranking	Inpatient palliative care (*n* = 276)			Acute hospital care (*n* = 276)		
	Drug class (ATC code)	n	%	Drug class (ATC code)	n	%
1	Natural opium alkaloids (N02AA)	233	84.4	Solutions affecting the electrolyte balance (B05BB)	268	97.1
2	Solutions affecting the electrolyte balance (B05BB)	221	80.1	Solutions for parenteral nutrition (B05BA)	223	80.8
3	Propulsives (A03FA)	152	55.1	Propulsives (A03FA)	200	72.5
4	Contact laxatives (A06AB)	146	52.9	Plain sulfonamide diuretics (C03CA)	198	71.7
5	Benzodiazepine derivatives (N05CD)	133	48.2	Electrolyte solutions (B05XA)	184	66.7
6	Solutions for parenteral nutrition (B05BA)	124	44.9	Contact laxatives (A06AB)	167	60.5
7	Glucocorticoids (H02AB)	123	44.6	Cephalosporins (J01DA)	167	60.5
8	Plain sulfonamide diuretics (C03CA)	118	42.8	Phenylpiperidine derivatives opioids (N02AB)	159	57.6
9	Butyrophenone derivatives antipsychotics (N05AD)	111	40.2	Natural opium alkaloids (N02AA)	156	56.5
10	Magnesium compounds (A02AA)	108	39.1	Adrenergic and dopaminergic agents (C01CA)	146	52.9

*ATC* Anatomical Therapeutic Chemical

### Medical costs

A comparison of the medical costs for both care groups is shown in Table 4. The combined daily medical costs were significantly lower for the inpatient palliative care group, compared to those for the acute hospital care group (adjusted mean = 107 US [101–113] dollars/day, vs. 253 [210–308] US dollars/day, respectively; *P* < 0.001). In addition, all daily medical cost categories were significantly higher in the acute hospital care group, including diagnoses, laboratory examinations, radiologic examinations, therapies, medications, and hemodialysis (all *P* < 0.005).

**Table 4:** Medical costs for terminal pancreatic cancer patients according to end-of-life care

Items	Inpatient palliative care (*N* = 276)		Acute hospital care (*N* = 276)		*P* ^*^
	Adjusted mean	95 % CI	Adjusted mean	95 % CI	
Diagnosis costs	11.5	11.2–12.0	12.9	12.3–13.5	0.001
Laboratory examination costs	5.9	4.9–6.9	26.8	22.8–33.4	<0.001
Radiologic examination costs	1.1	0.7–1.6	16.9	9.6–28.0	<0.001
Therapeutic costs	16.7	15.0–18.4	25.7	22.1–29.9	<0.001
Medication costs	25.4	22.4–29.6	61.8	54.8–68.9	<0.001
Hemodialysis costs	2.5	1.2–4.2	22.7	12.2–38.3	0.005
Total medical costs	107	101–113	253	210–308	<0.001

All values are reported as US dollars/day

^*^Analyzed using a generalized linear model with gamma distribution and log link function. 95 % CI: 95 % confidence interval

## Discussion

In this study, we collected comprehensive data from the NHI records of patients with terminal pancreatic cancer, and used these data to compare inpatient palliative care and acute hospital care. Our analysis revealed that the majority of patients with pancreatic cancer were men and had been hospitalized in medical centers. Furthermore, most patients were treated in acute hospital wards. In the inpatient palliative care group, approximately 40 % of patients were treated by family physicians, and these patients had shorter hospital stays, fewer aggressive procedures, and lower medical costs. Furthermore, these patients were prescribed more natural opium alkaloids and benzodiazepine derivatives, compared to the acute hospital care inpatients. However, given the relatively short hospital stays, we could not perform a detailed analysis of the durations for all medical procedures.

In Taiwan, the only available forms of hospice care are inpatient palliative care and home palliative care [17], and there were 50 hospital-based inpatient palliative care units and 69 home palliative care teams in April 2015 [18]. The palliative care teams only offer consultations for patients in acute hospital wards. The physicians in acute hospital wards are responsible for all care-related decisions for patients in acute hospital wards. It has been estimated that 13,000 patients with cancer receive these services each year in Taiwan [19], and inpatient palliative care is available and fully accessible to all healthcare beneficiaries, to whom it is offered at all medical centers and in select regional hospitals, although it is rarely offered in district hospitals. Furthermore, Taiwanese patients with pancreatic cancer are frequently referred to medical centers, which have a full complement of diagnostic facilities and greater treatment capabilities [20]. These patients can then be transferred to the inpatient palliative care units or acute hospital wards when they become terminally ill. Although sophisticated medical services are rarely used for patients who receive inpatient palliative care or acute hospital care, it is more convenient for patients at medical centers to access these services, compared to inpatients at regional or district hospital. Therefore, patients with terminal pancreatic cancer are more frequently admitted to medical centers, rather than to regional or district hospitals.

Inpatient palliative care is not widely used in Taiwan, and we found that the majority of terminally ill patients with pancreatic cancer elected to receive end-of-life care at acute hospital wards. Several factors might influence the decision to not select inpatient palliative care, such as physician preferences and referral practices, cultural concerns, individual patient choices and circumstances, and public or professional awareness of the benefits of inpatient palliative care [9, 10, 21, 22]. Unfortunately, in Taiwan, inpatient palliative care units are associated with a negative image (i.e., ‘death wards’), and a strong sense of familial obligation leads families to provide in-home care to sick family members. In addition, caregivers and family members typically prefer life-sustaining treatment for terminally ill patients, and some physicians prefer not to discuss end-of-life issues with their patients [23]. Therefore, these factors may motivate Taiwanese patients and their families to elect for end-of-life care in acute hospital wards, rather than in inpatient palliative care units.

The basic philosophical tenets of end-of-life care have been rooted in the recognition of an individual’s personal dignity. Therefore, the most common treatment objectives for inpatient palliative care include helping patients die with dignity, alleviating pain and suffering, controlling symptoms, and using less aggressive therapies [10, 24, 25]. In this study, we found that 84.4 % of the inpatient palliative care patients were prescribed natural opium alkaloids, which is noticeably higher than the prescription rate (72.7 %) in a previous study of inpatient palliative care for patients with hepatocellular carcinoma [17]. Furthermore, only 56.5 % of the patients in the acute hospital care group were prescribed natural opium alkaloids. However, among patients with pancreatic cancer, pain is a major source of distress [2], and adequate pain control is the primary priority in terminally ill cases, which may partially explain why the patients in the inpatient palliative care group more frequently received benzodiazepine derivatives (48.2 % vs. 26.8 % in the acute hospital care group). These drugs are an important adjuvant to control pain, and can help treat concomitant psychological disturbances, such as insomnia, anxiety, and depression, according to the World Health Organization’s guide for cancer pain relief [26]. Moreover, the patients in the inpatient palliative care group used fewer cephalosporins (32.2 % vs. 60.5 %, respectively) and adrenergic or dopaminergic agents (5.8 % vs. 52.9 %, respectively), which is likely because these treatments are typically futile in patients with terminal pancreatic cancer [27].

There were 4,686 deaths due to pancreatic cancer from 2003 to 2006 [28]. However, home palliative care is widely used in Taiwan [18], and many patients were discharged, against their physician’s advice, when they were dying [29]. Thus, only 854 patients with terminal pancreatic cancer who died in-hospital were included in this study. Nevertheless, our findings indicated that there were significant differences between inpatient palliative care and acute hospital care for patients with terminal pancreatic cancer.

Although we found that inpatient palliative care resulted in significantly shorter hospital stays, there is controversy in the existing literature regarding whether inpatient palliative care leads to shorter or longer hospital stays [7, 11]. In our study, patients in the palliative care units used fewer aggressive procedures, which may lead to shorter lifespans and shorter hospital stays. However, we also found that inpatient palliative care resulted in lower per-person or daily medical costs, compared to acute hospital care. These findings may be attributable to the patients’ poor general conditions after termination of anticancer treatment, and their rapidly growing pancreatic tumors. Moreover, the treatment of patients’ poor general condition and symptoms of pancreatic cancer are challenging, and typically empirical, in inpatient palliative care units. Therefore, increasing the use of solutions that affect electrolyte balance, solutions for parenteral nutrition, propulsives, electrolyte solutions, cephalosporins, and adrenergic or dopaminergic agents may prolong the lives of some patients. However, prolonging the lives of terminally ill patients, such as the patients in our study, will inevitably prolong their suffering. Therefore, as we found that inpatient palliative care cost less than acute hospital care, the cost-benefit ratio of acute hospital care should be subjected to further evaluation [25, 30, 31]. Furthermore, we only evaluated patients who were treated during 2003–2006, and the number of Taiwanese inpatient palliative care units has increased from 26 in 2004 to 53 in 2015 [32]. Therefore, inpatient palliative care has become more accepted by the general public, which further supports its consideration during end-of-life decision-making.

The major strength of this study was its nationwide population-based design, which included a relatively large number of patients with pancreatic cancer. Furthermore, this design facilitated a comprehensive evaluation of the medical behaviors and costs that were associated with inpatient palliative care and acute hospital care. Therefore, the findings of our study provide epidemiological evidence that inpatient palliative care provides a greater amount of palliative care for patients with terminally ill pancreatic cancer and is less expensive than acute hospital care. Thus, these findings may provide the basis for changing traditional Taiwanese perceptions regarding inpatient palliative care, and for promoting end-of-life inpatient palliative care for patients with pancreatic cancer.

This study has several limitations that should be considered when interpreting our findings. First, we could not obtain data regarding the patients’ educational and socioeconomic status, the preferences of the patients and their caregivers, patient life expectancies, and the attitudes of physicians toward inpatient palliative care. However, although only a limited number of covariates were included in the logistic regression model, our propensity-score matching at the patient level provided comparable baseline characteristics between the two groups, which may have eliminated some selection biases. Second, although fewer aggressive procedures and a lower cost burden are desirable factors, such as those observed in the inpatient palliative care group, we were unable to obtain data regarding the patients’ symptom burdens, pain scores, or quality of life measures. Therefore, future research should incorporate quality of life measures to advance our knowledge regarding the effects of inpatient palliative care. Third, eligible patients receive a fixed daily payment from Taiwan NHI for inpatient palliative care [33], and this payment may influence the observed differences in the use of medications, aggressive procedures, and incurred costs between the two groups.

## Conclusions

Inpatient palliative care units provided patients with pancreatic cancer more frequent pain control treatments, fewer aggressive procedures, and lower medical costs, compared to patients in acute hospital wards. Therefore, inpatient palliative care should be considered a viable option for treating patients with terminal pancreatic cancer.

## Abbreviations

NHI: National Health Insurance
ICD-9-CM: The International Classification of Diseases, 9th Revision, Clinical Modification
SD: standard deviation
ATC: Anatomical Therapeutic Chemical
95 % CI: 95 % confidence interval
